# MicroRNA dysregulation in cow's jejunum, jejunal lymph node, and caecal Peyer's patch during *Mycobacterium avium* subsp. *paratuberculosis* infection

**DOI:** 10.1186/s12864-025-12304-3

**Published:** 2026-01-08

**Authors:** Mengqi Wang, Nathalie Bissonnette, Pier-Luc Dudemaine, Eveline M. Ibeagha-Awemu

**Affiliations:** Agriculture and Agri-Food Canada, Sherbrooke Research and Development Centre, Sherbrooke, QC Canada

**Keywords:** Johne’s disease, MicroRNA, Immune response, Metabolism, Jejunum, Jejunal lymph node, Caecal Peyer’s patches

## Abstract

*Mycobacterium avium* subsp. *paratuberculosis* (MAP) is responsible for Johne’s disease (JD), a contagious granulomatous gastroenteritis with global distribution. This study investigated the expression of miRNAs and their potential regulatory mechanisms in Canadian Holstein cows with different MAP infection status, including 4 infected (MAPI), 5 tolerant to MAP infection (MAPT) and 5 healthy controls (HC).

Intestinal tissues, jejunum (JE), jejunal lymph node (JELN) and caecal Peyer’s patches (CPP) were collected for miRNA-sequencing, differential expression and functional analysis. A total of 318, 281 and 251 miRNAs were identified in JE, JELN, and CPP, respectively. Out of these, 28, 46, and 24 were highly expressed in JE, JELN and CPP, respectively, including 23 miRNAs highly expressed across all tissues. Their functional enrichment indicates overrepresentation in mostly immune related functions. More differentially expressed (DE) miRNAs were identified in CPP (39, 21 and 11) followed by the JELN (25, 8 and 13) and JE (9, 9 and 15) for the comparisons MAPI vs HC, MAPT vs HC, and MAPI vs MAPT, respectively. Six common DE miRNAs (bta-miR-125a, bta-miR-146a, bta-miR-146b, bta-miR-21-5p, bta-miR-320a, bta-miR-370) (FDR < 0.1) in the JELN and CPP of MAPI cows are implicated in the immune response, suggesting roles in MAP infection. Similarly, more functional annotations were recorded for the CPP (1284) than JELN (377) and JE (four). DE miRNAs in JELN and CPP were enriched in gene ontology (GO) terms (e.g. lymphocyte activation, lymphocyte homeostasis, leukocyte differentiation, regulation of cell differentiation, T cell receptor complex, alpha–beta T cell activation) and KEGG pathways (e.g. T/B cell receptor signaling pathways, Chemokine signaling pathway, Leukocyte transendothelial migration, Th17 cell differentiation, MAPK signaling pathway, etc.) with immune related functions indicative of participation in the regulatory mechanisms of the host immune response to MAP infection. The comparison MAPI vs HC revealed a more pronounced immune response in JELN and CPP indicative of a heightened immune response, but fewer DE miRNAs and less pronounced immune activation for MAPT vs HC indicative of the development of tolerance to MAP presence.

MiRNA expression and functional enrichment in JE, JELN, and CPP highlightes tissue-specific regulation of immune pathways and cellular functions during JD. Moreover, largely a different set of DE miRNAs, biological processes and pathways were driving the MAPI and MAPT phenotypes. These findings underscore the complex interplay between miRNAs and MAP in the studied tissues and provide a foundation for further exploration of miRNAs as potential biomarkers for the management of JD.

**Supplementary Information:**

The online version contains supplementary material available at 10.1186/s12864-025-12304-3.

## Introduction

Mycobacteria, the pathogenic agents of various human and animal diseases, are intracellular bacteria without motile ability, that have a bacterial morphology with thick and mycolic acid rich cell wall. *Mycobacterium avium* subsp. *paratuberculosis* (MAP) is one species of mycobacteria that mainly infect ruminants and other herbivores. In bovine, MAP causes paratuberculosis, commonly referred to as Johne’s disease (JD). JD is a contagious granulomatous gastroenteritis with global distribution. The clinical symptoms of JD at the advanced stage of MAP infection include diarrhea, submandibular edema, anemia, malnutrition, emaciation, and even death, leading to huge economic losses because of decreased productivity and premature culling [1]. Moreover, MAP is potentially zoonotic due to potential links with Crohn’s disease in humans [2, 3].

Shed MAP which occurs during the clinical stage of the infection, can survive in the environment for extended periods, such as in faeces, milk, bedding, food and water, leading to the infection of healthy animals and environment contamination [4, 5]. Owing to the lack of effective treatment drugs and vaccines, the most efficient strategy to control the transmission of JD is removal of the MAP infected animals from the herd during the subclinical stage when clinical signs are not visible. However, current MAP detection methods such as fecal culture (regarded as the “gold standard” for the detection of MAP presence) and methods like ELISA (detect MAP antibodies), have limited sensitivity to detect MAP [5, 6]. Therefore, strategies to enhance the sensitivity of current diagnostic tests will include deeper knowledge of the molecular mechanisms underlying JD and identification of biomarkers. Moreover, such knowledge will facilitate the breeding for JD resistance. Current estimates indicate that the heritability for JD susceptibility and antibody response to MAP range from 0.0389 to 0.186 [7–10], which is indication of the potential to control JD prevalence through breeding. Furthermore, a plethora of investigations have associated sequence variations in genes with immune functions and genomic regions with susceptibility [11–16] and resistance [17–19]to MAP infection. The identification of animals with genetic resistance or susceptibility to MAP infection based on biomarkers would provide a reference for the development of breeding strategies to reduce the incidence of JD.

Transcriptomic analysis is among strategies exploited to identify biomarkers [20, 21] to address mycobacterial infections. This technology (transcriptome mRNA) has been applied to study the host response to MAP infections in various tissues (blood, Peyer’s Patches, ileocecal valve, macrophages, jejunum/ileum and associated lymph nodes and salivary glands) [22–35]. MiRNAs, non-coding RNAs with short lengths (19–24 bp), play important regulatory roles by binding to target regions (mostly 3’ UTR) on mRNAs to impact their translation or promote decline of expressed transcripts [36, 37]. MiRNAs have been found to have effects on various cell processes since their initial discovery, especially the regulatory roles in host innate and adaptive immune responses to different disease pathogens [36, 38, 39]. The potential of miRNAs as biomarkers for human and various livestock diseases including JD has been reported [40–43]. In cattle, reports of altered miRNA expressions in the blood/serum [31, 44–47], intestinal tissues [29, 44], macrophages [48, 49], mammary epithelial cells [50], feces [51] and jejunum tissue [26] of MAP infected cattle suggests the involvement of miRNAs in immune related biological functions, such as inflammatory response, Toll-like receptor signaling, NF-kappa B signaling, JAK-STAT signaling, MAPK signaling, PI3K/Akt signaling, and Interleukin signaling pathways, etc.

The primary site of MAP infection is the gastrointestinal tract. In the ileum, MAP enters the ileal Peyer’s patches through M-cells and cause infection in local macrophages [52]. When the load of MAP bacteria turns to high level or the macrophages loss viability, MAP starts to be released. Released MAP bacteria also migrate into lymph nodes or neighboring intestinal cells or shed through feces [53]. MAP culture of different intestinal sites of MAP infected calves revealed that MAP was most frequently isolated from ileum and jejunum, and their corresponding lymph nodes [54]. Reports of gene expression and transcriptome alterations in MAP infected ileum and jejunum tissues or macrophages, suggests their important regulatory roles in the host immune response to JD [23, 25, 26, 29, 34, 35, 44, 48, 49, 55–58]. However, the miRNA alteration and roles in the regulation of the host immune response to MAP infection in the jejunum (JE), jejunal lymph node (JELN) and caecal Peyer’s patches (CPP) are still ambiguous.

Therefore, this study aimed to further understanding of the potential roles of miRNAs in response to MAP infection in the (JE, JELN and the CPP of cows with different MAP infection status, including MAP positive, MAP tolerant and healthy Canada Holstein cows.

## Material and methods

### Animal selection and JD diagnosis

Canadian Holstein cows were selected from commercial dairy farms with JD-positive cases in Quebec and Ontario, Canada. They were collected two times per year (every 5–7 months) by technicians or animal handlers under the supervision of a veterinarian, as reported previously [59]. Sampling and testing for the presence of MAP with three different methods including testing of MAP specific antibody in blood by Pourquier ELISA assay using the IDEXX MAP Ab test kit (IDEXX Laboratories, Markham, Ontario, Canada) (where S/P value > 55 is considered positive or indicate of a MAP-positive sample) and the test of MAP in fecal excretion by direct fecal qPCR (VetMAX™ Golf MAP Detection Kit) (where a Cq value for MAP DNA < 37 is considered positive) and mycobacterial culture were done as reported previously [60, 61]. Four cows positive for both serum ELISA and fecal culture (MAP +/+) constituted the MAP infected group (MAPI), five cows with positive results for serum ELISA but negative fecal culture results (MAP +/-) constituted the MAP tolerant group (MAPT), while five cows with negative results (MAP-/-) for both tests constituted the healthy control group (HC). The cows ranged in age from 3 to 7 years at the time of euthanasia. The 14 cows were purchased from the participating farms and housed in our barn (biosafety of level 2) for 1–3 weeks and euthanized humanely by intra-venous administration of 5 mg detomidin and 120 mL euthansol. JE, JELN and CPP tissues were collected, washed with phosphate buffered saline, cut into small pieces and immediately snap frozen in liquid nitrogen, and stored at −80 °C until used.

### RNA isolation

Tissue samples were removed from liquid nitrogen, weighed and 30 mg/sample was homogenized in 700 μL TRIzol Reagent (Life Technologies) using a Polytron homogenizer (Polytron PT 10–35 GT, Kinematica AG, Luzern, Switzerland) with a 7 mm probe for 10 s at 12,000 rpm, followed by total RNA extraction using miRNeasy Kit (Qiagen Inc., Toronto, ON, Canada). Total RNA was purified using Turbo DNA-free™ Kit (Ambion Inc. Foster City, CA, USA), the concentration was measured with Nanodrop ND-1000 (NanoDrop Technologies, Wilmington, DE, USA), and the integrity was measured using RNA 6000 Nano Labchip Kit (Agilent Technologies) on Agilent 2100 Bioanalyzer (Agilent Technologies, Santa Clara, CA, USA). Samples with RNA integrity number (RIN) ≥ 7.0 were used for miRNA-Seq library preparation.

### MiRNA library preparation and sequencing

The library preparation for small RNA sequencing was done as described in our previous study [62]. After adapter ligation and amplification, the desired libraries with inserted miRNA were separated by size selection based on polyacrylamide gel electrophoresis. And then, DNA clean and concentrator-5 kit (Zymo Research, Irvine, CA, USA) was used to concentrate the libraries, which were eluted from the gel. The Picogreen assay (Life Technologies, Waltham, MA, USA) and a Nanodrop 3300 fluorescent spectrophotometer (NanoDrop Technologies) were employed to measure the quality and quantity of purified libraries, and then qPCR was used to further validate the concentration of each library for Illumina platforms (KAPA Biosystems, Wilmington, MA, USA). Multiplexed libraries were sequenced on an Illumina HiSeq 2500 platform (single-end 50-base reads) by The Centre for Applied Genomics at The Hospital for Sick Children (Toronto, Canada).

### MiRNA sequence data analysis

Raw sequencing data was processed using the standard bioinformatics pipeline from nf-core (https://nf-co.re/smrnaseq) [63, 64]. Briefly, FastQC v0.12.0 was used to control the quality of raw sequence data, followed by trimming of adaptor sequences using Trim Galore! v0.6.5. Then Bowtie2 v2.5.0 was used to further clean the reads by removing contaminants and repeats. Clean reads were aligned to the bovine reference genome (ARS-UCD1.2, bosTau9) using bowtie v1.3.0.

### Known miRNA identification and novel miRNA discovery

The known miRNAs were identified by aligning the clean reads to mature miRNA from MiRBase v22.1 [65], and the novel miRNAs were discovered by using miRDeep2 (v2.0.1.2) [66]. Greater than 2 of MiRDeep2 score and a significant randfold *p* value were used to define the identification of potential novel miRNAs, which were then used to count the number of reads in JE, JELN and CPP. The known and novel miRNAs with ≥ 10 total reads in each of the three groups were considered as truly expressed miRNAs, and then used in next step analysis.

### Differential expression analysis

DESeq2 (v1.42.0) software was used for differential expression analysis of miRNAs. Comparisons were carried out between the normalized counts of miRNAs per tissue among groups, including MAPI (MAP +/+) vs HC (MAP -/-), MAPT (MAP +/-) vs HC (MAP -/-), and MAPI (MAP +/+) vs MAPT (MAP +/-). Significant differential expression (DE) of miRNAs between groups was defined as having a *p*-value < 0.05 and |log2 fold change (log2FC)|> 1.

### MiRNA target genes prediction and functional enrichment

TargetScanHuman8.0 [67] was employed to predict the target genes of differentially expressed (DE) miRNAs identified per tissue. The perl scripts (targetscan_70.pl and targetscan7_context_scores.pl) were carried out to predict the target genes for novel miRNAs. The target genes with cumulative weighted context + + score < −0.2 and context + scores ≥ 95th percentile were kept and further filtered by keeping only genes (mRNA) expressed in the same tissues from the same animals. The mRNA data has been reported previously [34]. Then the correlation analysis between DE miRNAs and target genes were processed by Spearman’s rank correlation coefficient, whose *p* values were adjusted by Benjamini–Hochberg procedure (false discovery rate, FDR) [68]. Target genes with significant correlations with their corresponding DE miRNA (|rho|> 0.3 and FDR < 0.05) were finally selected and used for gene ontology (GO) and KEGG pathways enrichment using ClueGO (v2.5.10) [69]. The FDR < 0.05 was used to define the significantly enriched GO terms and KEGG pathways. The assembly of GO terms and KEGG pathways into functional groups was assessed using the kappa score based on the shared genes. The overlap of enriched GO terms and KEGG pathways between comparison groups were visualized with the R packages “ggplot2” (v3.5.2) and “UpSet” (v1.4.0).

### Validation of miRNA expression by real time quantitative PCR (qPCR)

The expression level of three randomly selected DE miRNAs (bta-miR-125a, bta-miR-21-5p, and bta-miR-375) were validated by qPCR. The same total RNA from CPP tissues of cows in MAPT and control groups, which were used for miRNA sequencing, were reverse transcribed with the QuantiTect Reverse Transcription Kit from Qiagen (Qiagen, Toronto, Canada). The real time quantitative qPCR for each of the miRNAs was performed with miRCURY LNA SYBR® Green PCR Kits (Qiagen, Toronto, Canada) according to manufacturer’s recommendations and on a StepOne Plus System (Applied Biosystems, Foster City, CA, USA). UniSp6 was used to normalized the expression and the relative expression of miRNAs was calculated using the comparative Ct (∆∆Ct) method [70].

## Result

### Identification of miRNAs in response to MAP infection in cow intestinal tissues

Three cohorts of Canadian Holstein cows, exhibiting distinct conditions associated with MAP infection, were meticulously chosen for the purposes of this investigation. It is noteworthy to mention that one cow from the MAPT group lacked JELN tissue, and additionally, one cow from both the MAPT and HC groups lacked CPP tissues. Consequently, a total of 14 samples from JE (comprising 4 from MAPI, 5 from MAPT, and 5 from HC), 13 samples of JELN (comprising 4 from MAPI, 4 from MAPT, and 5 from HC), and 12 samples of CPP (four in each cohort) (Table S1) were subsequently subjected to miRNA profiling through small RNA sequencing (Table S2).

**Fig. 1:**
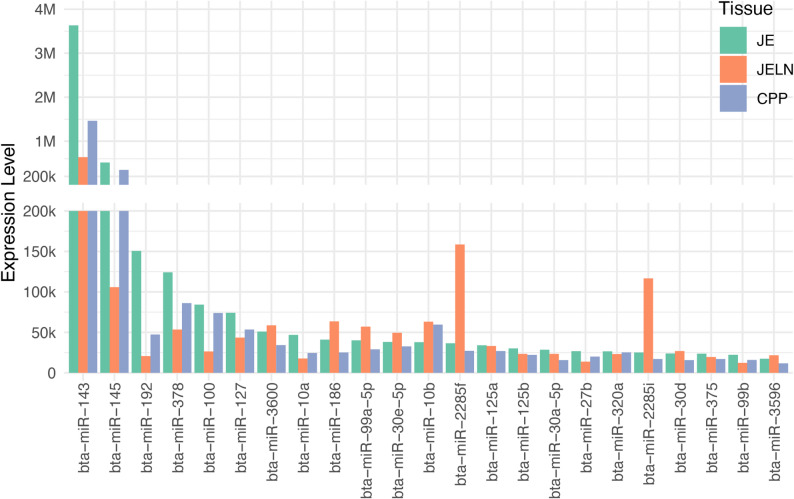
Twenty-three miRNAs highly expressed (average read counts per sample is greater than10,000) in the jejunum (JE), jejunal lymph node (JELN) and caecal Peyer’s patches (CPP). The miRNA expression levels shown are the average normalized read counts in the corresponding tissues

The miRNA profiling identified a total of 318, 281, and 251 known miRNAs and 130, 129, and 60 novel miRNAs in the JE, JELN and CPP tissues, respectively (Table S3A-D). Consequently, the cumulative tally of miRNAs identified amounted to 448, 410, and 311 in JE, JELN, and CPP, respectively (Table S3). MiRNAs with an average read count exceeding 10,000 per sample were considered as highly expressed, with 28, 46, and 24 such miRNAs identified in the JE, JELN and CPP, respectively (Table S3A-C). Notably, a subset of 23 miRNAs exhibited elevated expression across all three tissues, as outlined in Fig. 1 and Table S3E. Bta-miR-143 emerged as the most abundantly expressed across all three tissues, reaching its zenith in JE with 3,632,567 mean reads per sample, followed by CPP (1,462,719 mean reads per sample) and JELN (615,644 mean reads per sample). Furthermore, bta-miR-145 ranked as the second most abundantly expressed miRNA in both JE and CPP, with an average of more than 100 thousand reads per sample. In JELN, it held the fourth position, trailing bta-miR-143, bta-miR-2285f, and bta-miR-2285i. Additionally, bta-miR-192 and bta-miR-378 exhibited substantial expression levels in the JE, each surpassing 100 thousand reads per sample. Of the 23 highly expressed miRNAs, 15 demonstrated their peak expression in the jejunum, while 10 of them exhibited their lowest expression levels in JELN. Notable examples include bta-miR-143, bta-miR-145, bta-miR-192, bta-miR-378, bta-miR-100, bta-miR-127, bta-miR-10a, bta-miR-27b, bta-miR-320a, and bta-miR-99b. Conversely, 8 miRNAs displayed their highest expression in JELN, with 7 of them (bta-miR-3600, bta-miR-186, bta-miR-99a-5p, bta-miR-30e-5p, bta-miR-2285f, bta-miR-2285i, and bta-miR-30d) registering their lowest expression levels in CPP (Table S3E).

### Differentially expressed miRNAs associated with MAP infection

**Fig. 2:**
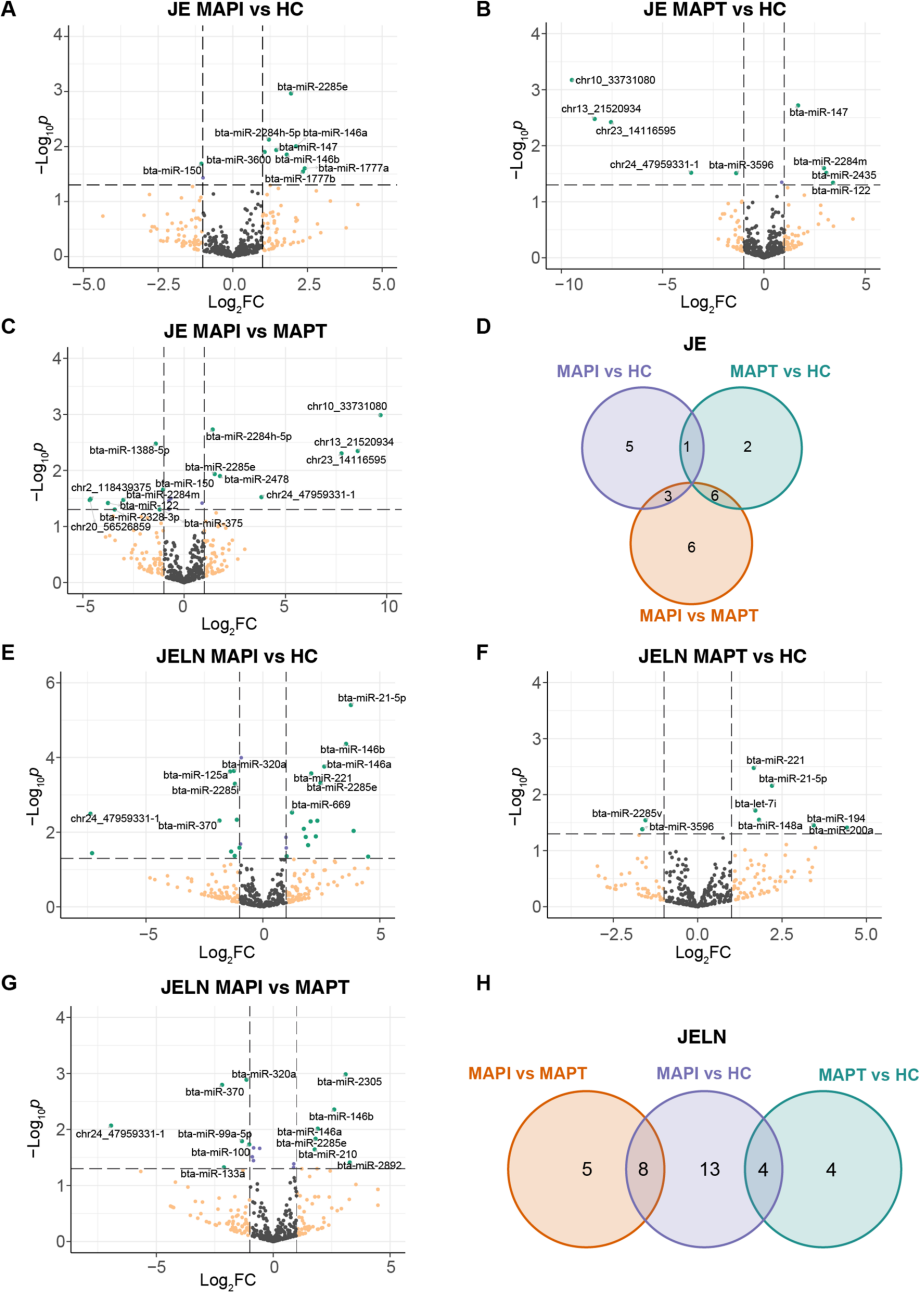
The expression status of miRNAs in the JE and JELN revealed DE miRNAs associated with MAP infection. **A-C** Volcano plots showing the DE miRNAs identified in the JE for the comparisons MAPI vs HC, *n* = 9 (**A**), MAPT vs HC, *n* = 9 (**B**) and MAPI vs MAPT, *n* = 15 (**C**). The DE miRNAs are represented by green dots with labeled names. **D** Venn diagram illustrating the intersecting DE miRNAs identified in the three comparisons within JE. **E–G** DE miRNAs identified in the JELN for the comparisons MAPI vs HC, *n* = 25 (**E**), MAPT vs HC, *n* = 8 (**F**) and MAPI vs MAPT, *n* = 13 (**G**), are donated by green dots with labeled name. **H** The intersecting DE miRNAs identified in the three comparisons within the JELN. DE: differential expression; JE: jejunum; JELN: jejunal lymph node; DE: differentially expressed; MAPI: MAP infected; MAPT: MAP tolerant; HC: healthy control; FC: fold change

The miRNAs expression levels were compared between the three groups per tissue (MAPI vs HC, MAPT vs HC and MAPI vs MAPT) to identify DE miRNAs associated with MAP infection. A total of 23 miRNAs showed significant differential expression (*P* < 0.05) between groups in JE, including 9, 9 and 15 miRNAs DE in the comparisons MAPI vs HC (Fig. 2A), MAPT vs HC (Fig. 2B) and MAPI vs MAPT (Fig. 2C), respectively (Table S3F). Only bta-miR-150 out of 9 DE miRNAs showed down-regulated expression level in MAPI vs HC group, while the other eight DE miRNAs were significantly upregulated. For instance, the expression of bta-miR-1777a, bta-miR-1777b and bta-miR-146a in MAPI group was more than four-fold higher than in HC group (log_2_FC > 2) (Fig. 2A). Some DE miRNAs were identified in more than one comparison (Fig. 2D). The up-regulated expression of bta-miR-147 was found in both MAPI (log_2_FC = 1.46) and MAPT (log_2_FC = 1.69) groups compared to HC group. Four out of five down-regulated DE miRNAs in MAPT group were novel miRNAs, including novelmiR_10_33731080, novelmiR_13_21520934, novelmiR_23_14116595, and novelmiR_24_47959331-1, whose expression levels were significantly higher in MAPI group compared to MAPT. Besides, bta-miR-122 and bta-miR-2284 m were identified as DE miRNAs in both MAPT vs HC (up-regulated) and MAPI vs MAPT (down-regulated) comparisons with significant higher expression in MAPT group compared to both MAPI and HC groups. Three DE miRNAs (bta-miR-150, bta-miR-2284 h-5p, and bta-miR-2285e) were identified in both MAPI vs HC and MAPI vs MAPT comparisons. Bta-miR-150 showed highest expression level in MAPT group, while the expression levels of bta-miR-2284 h-5p and bta-miR-2285e were significantly higher in MAPI group compared to MAPT and HC groups.

In the JELN, the comparisons MAPI vs HC, MAPT vs HC and MAPI vs MAPT revealed 25 (10 down- and 15 up-regulated) (Fig. 2E), 8 (2 down- and 6 up-regulated) (Fig. 2F), and 13 (7 down- and 6 up-regulated) (Fig. 2G) DE miRNAs (*P* < 0.05), respectively (Table S3F). Out of these, 14 DE miRNAs in the MAPI vs HC comparison remained significant after FDR correction (FDR < 0.1) (Table S3F). Fifteen out of 25 DE miRNAs from the comparison MAPI vs HC were up-regulated in MAPI group compared to HC group, four (bta-miR-221, bta-let-7i, bta-miR-148a, and bta-miR-21-5p) of which also showed significant higher expression levels in MAPT group than HC group (Fig. 2H). Eight DE miRNAs were both identified in the comparisons of MAPI vs HC and MAPI vs MAPT (Fig. 2H). For instance, four down-regulated DE miRNAs from the comparison of MAPI vs HC, including novelmiR_24_47959331-1, novelmiR_10_20364041-1, bta-miR-370 and bta-miR-320a, were also identified as down-regulated DE miRNAs between MAPI and MAPT group, and whose expression levels were lowest in MAPI group. On the contrary, four DE miRNAs (bta-miR-210, bta-miR-2285e, bta-miR-146a, and bta-miR-146b) were up-regulated in MAPI group compared to both MAPT and HC groups with highest and lowest expression levels in MAPI and HC, respectively. However, these eight DE miRNAs were not significantly differentially expressed between MAPT and HC group (Table S3E).

**Fig. 3:**
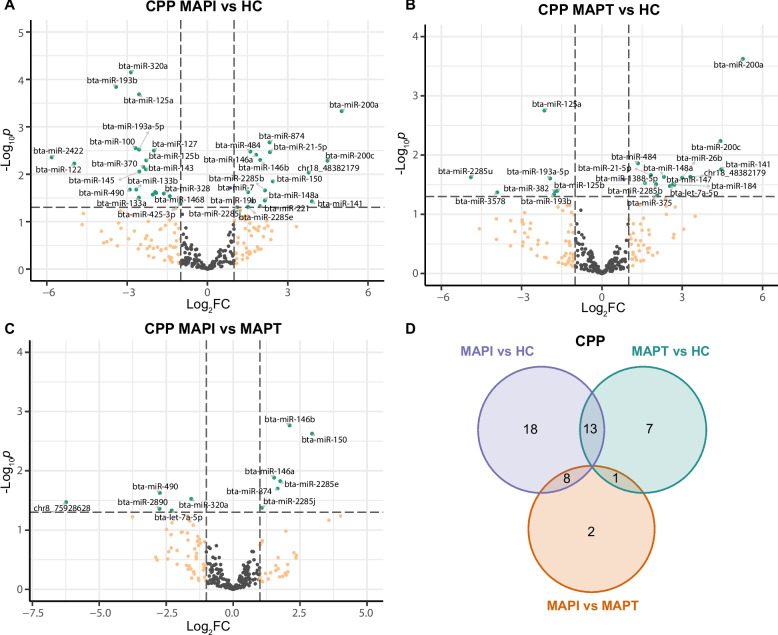
The expression status of miRNAs in the caecal Peyer’s patches revealed DE miRNAs associated with MAP infection. **A-C** Volcano plots showing the DE miRNAs identified in the comparisons MAPI vs HC, *n* = 39 (**A**), MAPT vs HC, *n* = 21 (**B**) and MAPI vs MAPT, *n* = 11 (**C**). The DE miRNAs are represented by green dots with labeled names. **D** Venn diagram illustrating the intersecting DE miRNAs identified in the three comparisons. DE: differential expression; MAPI: MAP infected; DE: differentially expressed; MAPT: MAP tolerant; HC: healthy control; FC: fold change

More DE miRNAs (*P* < 0.05) were identified in CPP, compromising 39 (22 down- and 17 up-regulated), 21 (7 down- and 14 up-regulated) and 11 (5 down- and 6 up-regulated) DE miRNAs in the comparisons MAPI vs HC, MAPT vs HC and MAPI vs MAPT, respectively (Figs. 3A-C, Table S3F). Out of these, 19 DE miRNAs in the MAPI vs HC comparison remained significant after FDR correction (FDR < 0.1) (Table S3F). Bta-miR-2422 (log_2_FC = −5.82), bta-miR-122 (log_2_FC = −4.97), and bta-miR-193b (log_2_FC = −3.41) were the three most down-regulated DE miRNAs in MAPI vs HC group. While bta-miR-200a (log_2_FC = 5.01), bta-miR-200c (log_2_FC = 4.49), bta-miR-141 (log_2_FC = 3.90) and novelmiR_18_48382179 (log_2_FC = 3.78) were the most up-regulated DE miRNAs in MAPI vs HC group (Fig. 3A, Table S3F). A total of 13 DE miRNAs were identified in both comparisons of MAPI vs HC and MAPT vs HC (Fig. 3D). The four most up-regulated DE miRNAs in MAPI vs HC were also ranked as the top four DE miRNAs with highest up-regulated expression in MAPT vs HC comparison (Fig. 3B), but no significant difference between MAPI vs MAPT (Fig. 3C). Another four (bta-miR-484, bta-miR-21-5p, bta-miR-2285b and bta-miR-148a) and five (bta-miR-125a, bta-miR-193a-5p, bta-miR-193b, bta-miR-382 and bta-miR-125b) DE miRNAs were also identified as up-regulated and down-regulated in the comparisons MAPI vs HC and MAPT vs HC, respectively, but with no significant differences between MAPI vs MAPT groups. All six up-regulated DE miRNAs identified in MAPI vs MAPT groups also showed significantly higher expression in MAPI group than HC group. In addition, bta-miR-320a and bta-miR-490 were identified as down-regulated DE miRNAs in MAPI vs MAPT and MAPI vs HC comparisons, with highest expression levels in HC group. Bta-let-7a-5p was the only DE miRNA identified in both MAPI vs MAPT and MAPT vs HC comparisons, and which showed significantly higher expression level in MAPT group than both MAPI and HC groups.

Interestingly four miRNAs showed significant differential expression (*P* < 0.05) levels across groups in all three tissues, including bta-miR-146a, bta-miR-146b, bta-miR-2285e and bta-miR-122. JELN and CPP shared more common DE miRNAs, including bta-miR-125a, bta-miR-320a, bta-miR-21-5p, bta-miR-146a, bta-miR-146b, bta-miR-370 (FDR < 0.1), bta-miR-148a, bta-miR-221, bta-miR-122 and bta-miR2285e (*P* < 0.05) (Table 1) for MAPI vs HC comparison, bta-miR-148a, bta-miR200a and bta-miR-21-5p for MAPT vs HC comparison and bta-miR-146a, bta-miR-146b, bta-miR-2285e and bta-miR-320a for the MAPI vs MAPT comparison (Table S3F).

**Table 1:** Common differentially expressed miRNAs in jejunum lymph node and caecal Peyer’s patches of *Mycobacterium avium* subsp. *Paratuberculosis* infected cows

miRNA	jejunum lymph node			Faecal Peyer’s patches		
	Log2 fold change	*P*value	FDR	Log2 fold change	*P*value	FDR
bta-miR-122	3.880	0.0092	0.1580	−4.974	0.0059	0.0813
bta-miR-125a	−1.403	0.0002	0.0111	−2.551	0.0002	0.0142
bta-miR-146a	2.625	0.0002	0.0111	1.821	0.0039	0.0727
bta-miR-146b	3.558	4.32E-05	0.0063	1.972	0.005	0.0760
bta-miR-148a	2.328	0.0049	0.0996	2.144	0.0361	0.2187
bta-miR-21-5p	3.764	3.94E-06	0.0011	2.337	0.0034	0.0703
bta-miR-221	2.068	0.0003	0.0111	1.974	0.0469	0.2602
bta-miR-2285e	2.461	0.0005	0.0163	1.513	0.0489	0.2622
bta-miR-320a	−1.241	0.0002	0.0111	−2.859	7.09E-05	0.0142
bta-miR-370	−1.858	0.0049	0.0996	−2.394	0.0069	0.0894

Bta-let-7b and novelmiR_24_47959331-1 were identified as DE miRNAs in both JE and JELN. In addition, bta-miR-150, bta-miR-147, bta-miR-375, and bta-miR-1388-5p were identified as DE miRNAs in JE or JELN and CPP (Table S3F).

### Predicted target genes of highly expressed and DE miRNAs

The target genes of DE miRNAs predicted with TargetScan were further filtered against mRNAs expressed in the same tissues under the same experimental conditions [34]. Only target genes whose mRNA expression levels showed at least moderate correlation (|*r*|> 0.3 and FDR < 0.05) with the corresponding DE miRNA in the same tissue were kept as the final predicted target genes for the next steps (Table S4). A total of 615, 422 and 985 target genes were predicted for 28, 46 and 24 highly expressed miRNAs in JE, JELN and CPP, respectively (Table S4A-C).

A total of 115, 160 and 27 target genes were predicted for the DE miRNAs in JE for the comparisons MAPI vs HC, MAPT vs HC and MAPI vs MAPT, respectively (Table S4D-F). Notably, the mRNA expression levels of six (out of 18) predicted target genes, including *FAM206A*, *GFM2*, *HYLS1*, *MPHOSPH6*, *SPRTN*, and *TSG101*, exhibited strong negative correlations with the expression level of bta-miR-2284h-5p in JE. Bta-miR-2284h-5p was also identified as DE in JE in both MAPI vs HC and MAPI vs MAPT comparisons. In addition, bta-miR-2478, an up-regulated DE miRNAs in MAPI vs MAPT group, also had significant strong negative correlation with three of its target genes (*CD28*, *DNTTIP1*, and *SMOX*).

Within JELN, 529 target genes were predicted for the 25 DE miRNAs between MAPI vs HC group (Table S4G). Bta-miR-103 had the most target genes (*n* = 64), out of which *MYBL1* was nearly perfectly negatively correlated with it (rho = −0.95). Fifty four target genes were predicted for a novel DE miRNA (novelmiR_13_39567358), which was down-regulated in MAPI vs HC group. Remarkably, 11 target genes (*ADO*, *CEBPG*, *FAM102B*, *FBXO30*, *GXYLT1*, *IPMK*, *SAR1A*, *SPRYD7*, *TGIF1*, *TMEM65*, and *ZNRF2*) with higher expression levels in MAPI group than HC group exhibited significant negative correlations with the expression level of novelmiR_13_39567358*.* Besides, the down-regulated DE bta-miR-2285i was significantly negatively and strongly correlated with four of its target genes (*CBR4*, *CPT2*, *DYNLT1*, and *YBX1*). While the most up-regulated DE miRNA (bta-miR-2332) in MAPI vs HC had significant strong negative correlation with five of its target genes (*LIG4*, *NUDT15*, *SLC35A1*, *TRUB1*, and *USP45)*. In addition, bta-miR-452, bta-miR-92a, and bta-miR-296-3p, etc., also exhibited strong negative correlations with some of their target genes in cows of MAPI vs HC group (Table S4G). In the comparison MAPI vs MAPT, 161 target genes were predicted for the DE miRNAs with bta-miR-320a having the most target genes (*n*= 42) (Table S4H). Notably, bta-miR-320a, the DE miRNAs identified in both MAPI vs HC and MAPI vs MAPT comparisons, was significantly correlated with its six target genes, including *BRI3*, *IPO5*, *PSPH*, *TFRC*, *USP12*, and *VDAC1*. Similarly, bta-miR-210, upregulated DE miRNAs in MAPI vs HC and MAPI vs MAPT groups, had 21 predicted target genes and exhibited strong negative correlations with *BCAR3*, *CCDC24*, *MSX1*, and *SMIM5*. Totally 186 target genes were predicted for the DE miRNAs identified between MAPT vs HC group, with bta-miR-148a having the most target genes (*n* = 41) (Table S4I). Only bta-let-7i, bta-miR-3596, bta-miR-2285v and bta-miR-21-5p had strong negative correlations with one of their target genes, *DDX26B*, *SLAMF8*, *SDR39U1*, *ALG13*, respectively.

More target genes were predicted for DE miRNAs in CPP, compromising 1565, 901 and 334 target genes for the DE miRNAs identified in the comparisons MAPI vs HC, MAPT vs HC and MAPI vs MAPT, respectively (Table S4J-L). In the MAPI vs HC comparison, the highest number of target genes (*n* = 168) were predicted for the up-regulated bta-miR-7. Among them, 61 demonstrated significant negative and strong correlations with bta-miR-7 (Table S4J). Following, bta-miR-125b, down-regulated in both MAPI vs HC and MAPT vs HC groups had 139 predicted target genes with strong negative correlations with 52 of them (Table S4J-K). Similarly, 91 86, 28 and 5 target genes were predicted for bta-miR-125a, bta-miR-193b, bta-miR-193a-5p and bta-miR-382, respectively. Amongst them, 28, 33, 3 and 2 showed strong negative correlations with bta-miR-125a, bta-miR-193b, bta-miR-193a-5p and bta-miR-382, respectively (Table S4J-K). Besides, 13 (out of 117) and 31 (out of 80) target genes exhibited strong negative correlations with bta-miR484 and novelmiR_18_48382179, respectively. The up-regulated DE miRNAs identified in both MAPI vs HC and MAPT vs HC comparisons, including bta-miR-2285b, bta-miR-21-5p, bta-miR-200a, bta-miR-200c, and bta-miR-141, were also significantly strongly negatively correlated with more than three of their target genes (Table S4J-K). In addition, strong negative correlations were also observed between the up-regulated DE miRNAs (bta-miR-150, bta-miR-146a, bta-miR-146b, bta-miR-2285j, and bta-miR-874) and down-regulated DE miRNAs (bta-miR-490 and bta-miR-320a) identified in both MAPI vs HC and MAPI vs MAPT and at least three of their target genes (Table S4J and L). In summary, the target genes exhibiting robust negative correlations with DE miRNAs underscored their considerable potential for regulation by these miRNAs. This observation suggests that these genes may actively contribute to the intricate network of host responses during MAP infection.

### Functional enrichment for the target genes of highly expressed and DE miRNAs

The target genes of highly expressed miRNAs in the JE (*n* = 28) were significantly enriched in 248 gene ontology (GO) terms (213 biological processes [BP], 7 molecular functions [MF], and 28 cellular components [CC]) and 15 KEGG pathways, primarily associated with immune-related processes and functions (Table S5A). The two largest and most significant BP-GO term groups, "Group66" (33 BP terms) and "Group65" (29 BP terms), were predominantly linked to immune system regulation, including terms such as the regulation of immune system processes (GO:0002682), leukocyte activation (GO:0045321), regulation of response to stimulus (GO:0048583), and lymphocyte activation (GO:0046649) (Table S5A). This highlights the central role of immune response regulation in JE, underscoring the importance of miRNAs in modulating these pathways.

In contrast, the 422 predicted target genes of highly expressed miRNAs in the JELN (*n* = 46) were enriched in fewer functional annotations including 48 BP-GO terms, 8 MF-GO terms, and 3 KEGG pathways (Table S5B). Despite fewer enriched categories, these annotations still pointed to key immune functions, including leukocyte activation involved in immune response (GO:0002366), positive regulation of lymphocyte activation (GO:0051251), myeloid leukocyte differentiation (GO:0002573), and positive regulation of leukocyte differentiation (GO:1,902,107). Additionally, significant enrichment was observed in GO terms related to carboxylic acid metabolism (GO:0019752) and hematopoiesis (GO:0030097), further indicating the role of miRNAs in immune cell differentiation and metabolism.

For the CPP, the 985 target genes predicted for its 24 highly expressed miRNAs were significantly enriched in 419 GO terms and 12 KEGG pathways (Table S5C), mostly immune processes reflecting a broader range of immune-related functions. Notable processes included the regulation and activation of various immune cell types, such as CD4-positive alpha–beta T cell differentiation involved in immune response (GO:0002294), T-helper 17 cell lineage commitment (GO:0072540), leukocyte activation (GO:0045321), T cell proliferation (GO:0042098), and B cell proliferation (GO:0042100) (Table S5C). These findings highlight the extensive involvement of the highly expressed miRNAs in regulating immune cell differentiation, activation, and proliferation in the three tissues, suggesting their critical roles in the immune response to MAP infection.

The target genes of the DE miRNAs identified in JE were barely enriched in functional annotations. Only the target genes of the 15 DE miRNAs identified in JE MAPI vs MAPT comparison were significantly enriched in one biological process (BP)-GO term (alpha-amino acid biosynthetic process, GO:1,901,607. FDR = 0.004) and 3 KEGG pathways (Intestinal immune network for IgA production (KEGG:04672, FDR = 0.011), N-Glycan biosynthesis (KEGG:00510, FDR = 0.011) and Various types of N-glycan biosynthesis (KEGG:00513, FDR = 0.010)) (Table S5D).

**Fig. 4:**
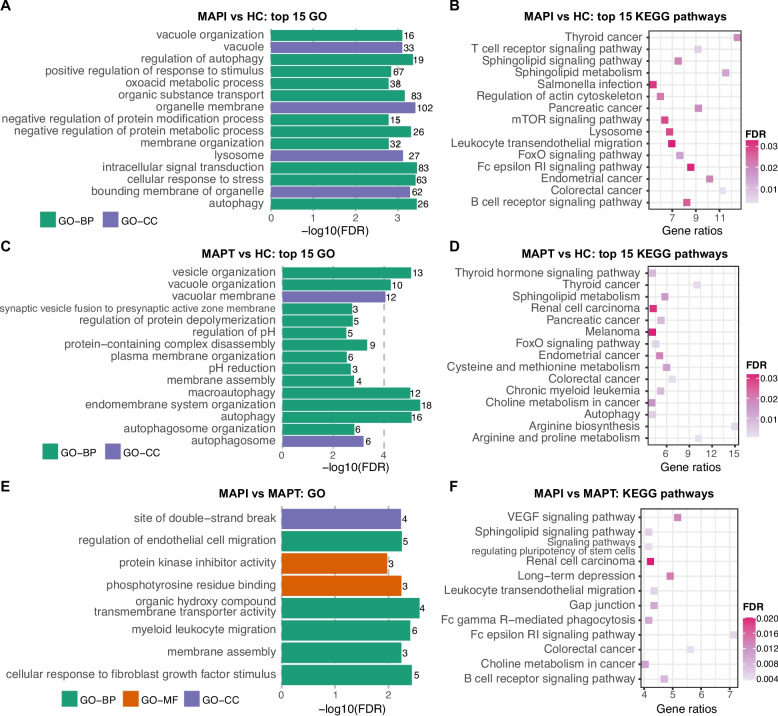
Gene ontology (GO) terms and KEGG pathways significantly enriched by target genes of DE miRNAs identified in jejunal lymph node. **A** and **C** shows the top 15 GO terms most significantly enriched by target genes of DE miRNAs identified in the comparisons MAPI vs HC (**A**) and MAPT vs HC (**C**). **B** and **D** shows the top 15 KEGG pathways most significantly enriched by target genes of DE miRNAs identified in the comparisons MAPI vs HC (**B**) and MAPT vs HC (**D**). **E** Eight GO terms and (**F**) 14 KEGG pathways significantly enriched by target genes of DE miRNAs identified in the comparison MAPI vs MAPT. The number at the right side of each bar in (**A**), (**C**) and (**E**) represents the count of target genes involved in the corresponding GO terms. DE: differentially expressed; MAPI: MAP infected; MAPT: MAP tolerant; HC: healthy control; FDR: false discovery rate; GO-BP: Gene ontology terms in the category of Biological Process; GO-MF: GO terms in the category of Molecular Function; GO-CC: GO terms in the category of Cellular Component

The target genes of the DE miRNAs identified in JELN showed abundant enrichment in functional annotations. The 25 DE miRNAs (529 target genes) of the MAPI vs HC comparison were significantly enriched in 279 GO term comprising 228 BP-, 23 MF- and 28 CC- GO terms, and 25 KEGG pathways (Table S5E). As showed in Fig. 4A, the top three GO terms with greatest significance were intracellular signal transduction (GO:0035556, FDR = 3.58 × 10^–4^), autophagy (GO:0006914, FDR = 3.58 × 10^–4^) and cellular response to stress (GO:0033554, FDR = 3.88 × 10^–4^), showing close relation with immune functions. In addition, a large proportion of significantly enriched KEGG pathways are associated with the immune responses (Fig. 4B, Table S5E). Besides, the 304 functional annotations were clustered into 82 groups, with 30 groups containing no less than 5 GO terms and/or KEGG pathways. The biggest cluster (Group81) included 27 BP-GO terms related to the immune response, such as innate immune response-activating signaling pathway (GO:0002758, FDR = 7.71 × 10^–3^), cytoplasmic pattern recognition receptor signaling pathway (GO:0002753, FDR = 0.011), and negative regulation of immune system process (GO:0002683, FDR = 0.020), among others (see “group81” in Table S5E). The cluster, “Group80”, included 25 BP-GO terms related to transmembrane transport and homeostasis, while “Group79” is a cluster of 15 KEGG diseases pathways and 2 BP-GP terms related to immune functions (Table S5E). In addition, “group78” emerged as the second biggest cluster with 14 BP- and 3 CC-GO terms related to catabolic process, autophagy and related regulation (see “group78” in Table S5E).

The DE miRNAs identified in JELN from the comparison MAPT vs HC were significantly enriched in 30 BP-, one MF-, four CC-GO terms and 16 KEGG pathways (Table S5F). Endomembrane system organization (GO:0010256, FDR = 3.83 × 10^–6^) and Arginine and proline metabolism (KEGG:00330, FDR = 5.61 × 10^–4^) were the most significant GO term and KEGG pathway, respectively (Figs. 4C-D). The 51 functional annotations were clustered into 17 groups, 6 of which contain at least 5 GO terms and/or KEGG pathways (Table 5 F). Ten disease-related KEGG pathways and one MF-GO term constituted the biggest group (“Group 16”) (see “Group16” in Table S5F). The second biggest group (“Group15”) consisted of 5 BP-, 2 CC-GO terms and 2 KEGG pathways related to autophagy (see “Group15” in Table S5F). While “Group14” included 7 BO-GO terms related to exocytic process, “Group12” comprised 5 BP- and 1 CC-GO term related to autophagosome maturation (Table S5F) and “Group11” cluster comprised 3 BP-GO terms and 2 KEGG pathways related to metabolic process, including Arginine and proline metabolism (KEGG:00330, FDR = 5.61 × 10^–4^), Arginine biosynthesis (KEGG:00220, FDR = 2.28 × 10^–3^), amine biosynthetic process (GO:0009309, FDR = 3.19 × 10^–3^), Cysteine and methionine metabolism (KEGG:00270, FDR = 0.014) and biogenic amine metabolic process (GO:0006576, FDR = 0.018) (See “Group11” in Table S5F).

A total of 8 GO (5 BP-, 2 MF- and 1 CC-) terms and 14 KEGG pathways were significantly enriched by the target genes of DE miRNAs identified in MAPI vs MAPT comparison in the JELN (Figs. 4E-F, Table S5G). These GO terms and KEGG pathways were clustered into 8 groups, with only one of them (Group 7) having more than 5 annotations. The “Group7” comprised 14 KEGG pathways, 5 BP-, 2 MF- and 1 CC-GO terms with disease and immune related functions, such as B cell receptor signaling pathway (KEGG:04662, FDR = 8.04 × 10^–3^), Leukocyte transendothelial migration (KEGG:04670, FDR = 5.23 × 10^–3^), Colorectal cancer (KEGG:05210, FDR = 7.92 × 10^–3^), and Sphingolipid signaling pathway (KEGG:04071, FDR = 5.66 × 10^–3^) amongst others (Table S5G).

**Fig. 5:**
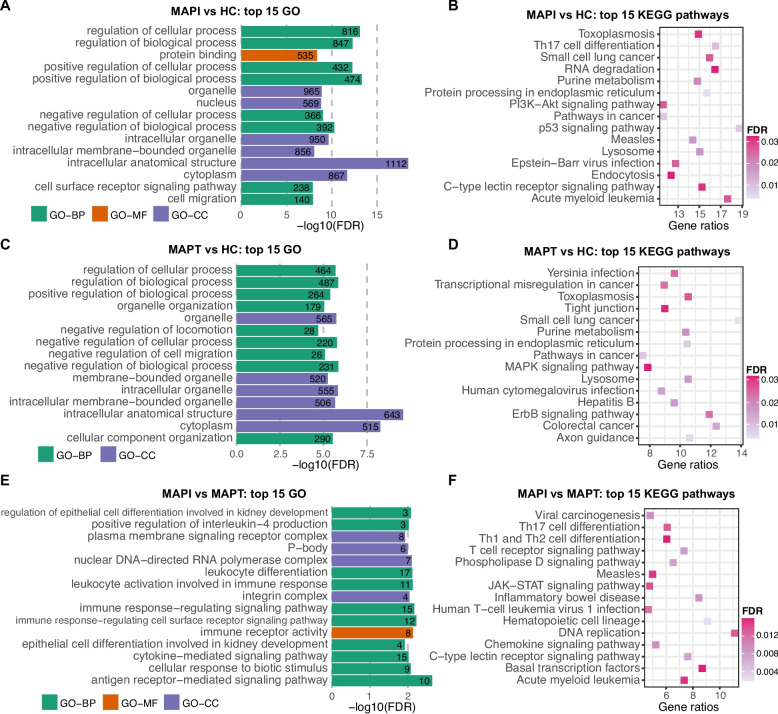
Gene ontology (GO) terms and KEGG pathways significantly enriched by target genes of DE miRNAs identified in caecal Peyer’s patches. **A**, **C** and **E** shows the top 15 GO terms most significantly enriched by the target genes of DE miRNAs identified in the comparisons MAPI vs HC (**A**), MAPT vs HC (**C**) and MAPI vs MAPT (**E**). The number at the right side of each bar in (**A**), (**C**) and (**E**) represents the count of target genes involved in the corresponding GO terms. **B**, **D** and **F** shows the top 15 KEGG pathways most significantly enriched by target genes of DE miRNAs identified in the comparisons MAPI vs HC (**B**), MAPT vs HC (**D**), and MAPI vs MAPT (**F**). DE: differentially expressed; MAPI: MAP infected; MAPT: MAP tolerant; HC: healthy control; FDR: false discovery rate; GO-BP: Gen ontology terms in the category of Biological Process; GO-MF: GO terms in the category of Molecular Function; GO-CC: GO terms in the category of Cellular Component

The target genes of the DE miRNAs identified in CPP were significantly enriched in more GO terms and KEGG pathways compared to both JE and JELN. For instance, the target genes of DE miRNAs in CPP identified from the comparison of MAPI vs HC were significantly enriched in 655 functional annotations, including 520 BP-, 60 CC-, 54 MF-GO terms and 21 KEGG pathways (Figs. 5A-B, Table S5H). The functional annotations were clustered into 181 groups, including 70 groups containing no less than 5 GO terms and/or KEGG pathways (Table S5H). Notably, the largest group (“Group180”) comprised 94 BP- and 1 MF-GO terms related to important processes and regulations involved in immune defense (see “Group180” in Table S5H), such as regulation of response to stimulus (GO:0048583, FDR = 1.52 × 10^–7^), T cell differentiation (GO:0030217, FDR = 2.29 × 10^–5^), leukocyte activation involved in immune response (GO:0002366, FDR = 3.81 × 10^–5^), leukocyte differentiation (GO:0002521, FDR = 6.13 × 10^–5^), amongst others (Table S5H). While the second largest group (“Group179”) included 82 BP-, 8 CC- and 3 MF-GO terms related to biological and cellular processes, particularly metabolic process such as positive regulation of biological process (GO:0048518, FDR = 5.15 × 10^–14^), regulation of cellular process (GO:0050794, FDR = 7.82 × 10^–14^), positive regulation of metabolic process (GO:0009893, FDR = 5.19 × 10^–8^), regulation of metabolic process (GO:0019222, FDR = 1.35 × 10^–5^), etc. (see “Group179” in Table S5H). Meanwhile, the third largest group was constituted by 89 BP-GO terms related to cell migration and tissue development (see “Group178” in Table S5H).

The DE miRNAs target genes of the MAPT vs HC comparison in CPP showed significant representation in 428 BP-, 50 CC-, 36 MF-GO and 25 KEGG pathways, which were further clustered into 161 groups (Figs. 5C-D, Table S5I). Amongst them, 56 groups contained no less than 5 functional annotations. The “Group160” was the most significant and largest group made up of 120 BP-GO terms and 1 KEGG pathway related to tissue development and cellular activities, notably cell proliferation and differentiation (Table S5I). The second largest group (“Group159”) contained 63 BP- and 8 CC-GO terms mainly related to the regulation of metabolic processes and biosynthetic process (See “Group159” in Table S5I) while the third largest group (“Group158”) contained 43 BP-, 2 CC- and 3 MF-GO terms related to cellular component organization or biogenesis (See “Group158” in Table S5I).

Besides, a total of 64 GO terms and 26 KEGG pathways were significantly enriched by the target genes of DE miRNAs identified in CPP through the MAPI vs MAPT comparison (Figs. 5E-F, Table S5J). The functional annotations were further clustered into 28 groups, including 7 groups having at least 5 annotations. The most significant group (“Group27”) which is also the largest consisted of 22 BP-GO terms and 2 KEGG pathways with related immune functions, such as antigen receptor-mediated signaling pathway (GO:0050851, FDR = 2.31 × 10^–3^), immune response-regulating cell surface receptor signaling pathway (GO:0002768, FDR = 6.00 × 10^–3^), immune response-regulating signaling pathway (GO:0002764, FDR = 6.77 × 10^–3^), leukocyte activation involved in immune response (GO:0002366, FDR = 7.44 × 10^–3^), etc. (Table S5J).

**Fig. 6:**
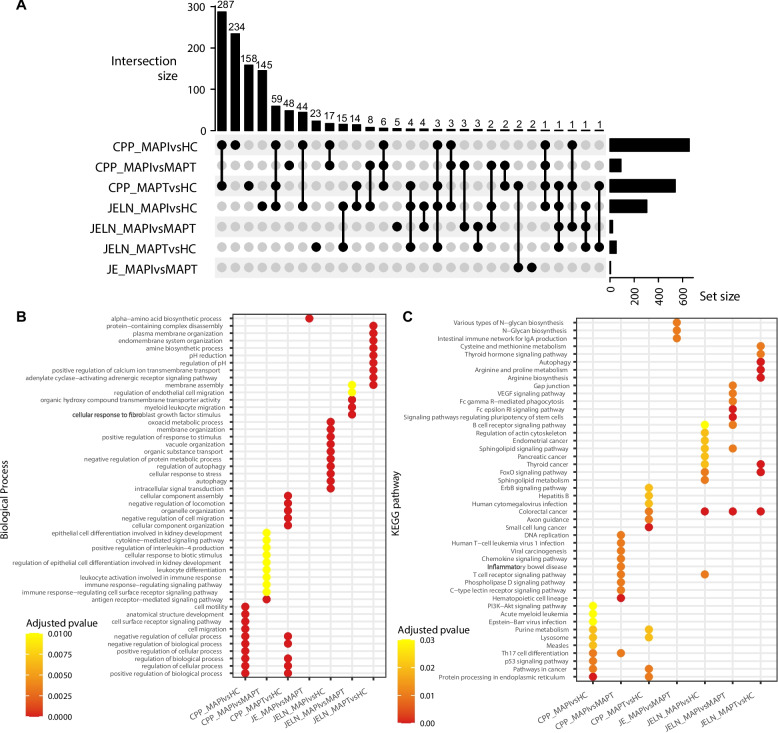
Comparison of enriched biological processes and pathways across experimental groups. **A** UpSet plot showing the intersection of enriched functional terms across different comparison groups. Each set represents pathways and biological processes enriched in a specific group, and the bars indicate the number of terms unique or shared among groups. **B**-**C** Bubble plot illustrating the top 10 enriched Biological Processes (**B**) and KEGG pathways (**C**) per comparison group based on adjusted *p*-values. Dot color indicates the adjusted *p* value. Functional terms are shown on the y-axis and comparison groups on the x-axis

An analysis of the overlap of enriched functional terms between comparison groups indicated that the majority of significant GO terms and KEGG pathways were uniquely enriched in each comparison or two comparisons within the same tissue, with only a small proportion shared between two or more comparisons across tissues (Fig. 6A). For example, 287 functional terms were unique to MAPI vs HC and MAPT vs HC in CPP, 234 were unique to MAPI vs HC in CPP, 158 were unique to MAPT vs HC in CPP and 145 were unique to MAPI vs HC in JELN. Interestingly, 3 terms (autophagy [GO:0006914], autophagosome [GO:0005776] and macroautophagy [GO:0016236]) were unique to two comparisons in two tissues, MAPI vs HC and MAPT vs HC in CPP and JELN. One term, leukocyte differentiation (GO:0002521) was enriched in four comparisons (MAPI vs HC, MAPI vs MAPT and MAPT vs HC in CPP, and MAPI vs HC) in JELN and another term, colorectal cancer pathway (KEGG:05210) was also enriched in four comparisons (MAPT vs HC in CPP, MAPI vs HC, MAPI vs MAPT and MAPT vs HC) in JELN. The JE with the least number of enriched terms, shared two of its four enriched terms (N-glycan biosynthesis [KEGG:00510] and various types of N-glycan biosynthesis [KEGG:00513]) between its MAPI vs MAPT comparison and MAPT vs HC comparison in the CPP. The top 10 enriched terms, many of which have functions in immune and disease processes, were unique to each comparison pairs and tissue with few terms shared between comparisons within tissues and even fewer across tissues (Figs. 6 B-C). For instance, five GO-BP terms (positive regulation of biological process (GO:0048518), regulation of cellular process (GO:0050794), positive regulation of cellular process (GO:0048522), negative regulation of biological process (GO:0048519) and negative regulation of cellular process (GO:0048523)) and four pathways (pathways in cancer (KEGG:05200), protein processing in endoplasmic reticulum (KEGG:04141), lysosome (KEGG:04142) and purine metabolism (KEGG:00230)) were common to the MAPI vs HC and MAPT vs HC comparisons in the CPP, while colorectal cancer pathway (KEGG:05210) was common to four comparisons in CPP and JELN.

### Quantitative real time PCR (qRT-PCR) validation of miRNAs expression

**Fig. 7:**
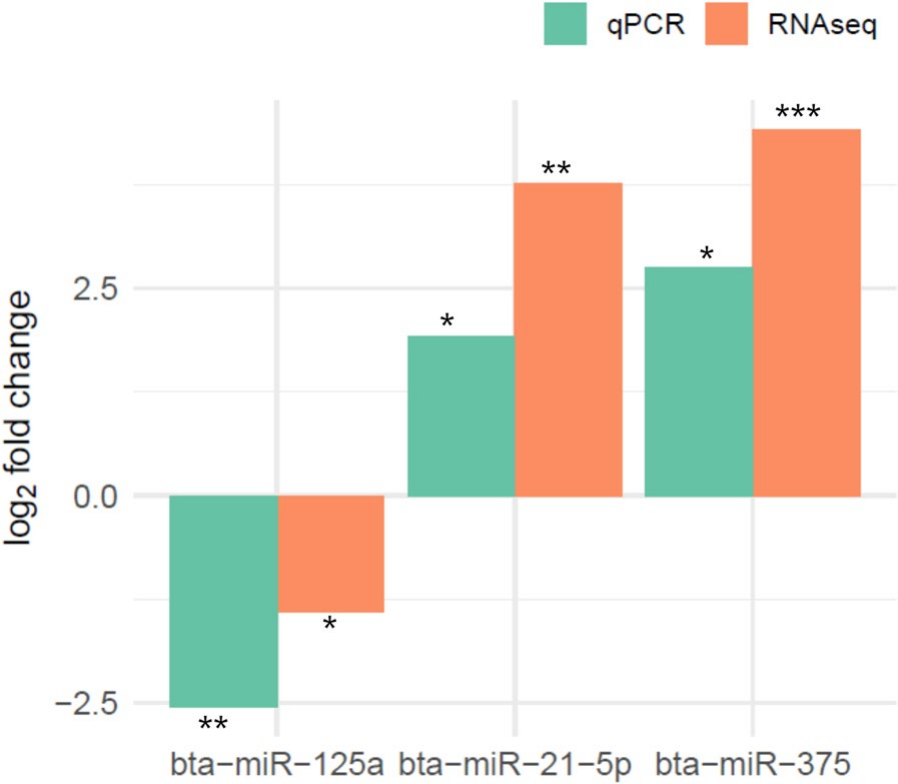
Relative expression of three miRNAs by RNA-seq and qPCR. The miRNAs were upregulated (bta-miR-21-5p and bta-miR-375) or downregulated (bta-miR-125a) by both the methods of RNA-seq and qPCR. The green and orange bars represent the relative expression changes of corresponding miRNAs between MAPI and HC groups detected by qPCR and RNA sequencing in caecal Peyer’s patches

To validate the expression results obtained by RNA-sequencing, the expression levels of bta-miR-125a, bta-miR-21-5p, and bta-miR-375 were measured by qPCR. The qRT-PCR detected significantly lower expression level of bta-miR-125a, and higher expression levels of bta-miR-21-5p and bta-miR-375 in CPP tissues of MAPT cows compared to HC cows (Fig. 7), which is consistent with the RNA-seq results.

## Discussion

*Mycobacterium avium* subsp. *paratuberculosis* primarily infect and persist within macrophages, particularly in the intestine, where they evade immune responses and establish chronic infection. The bacteria (MAP) predominantly invades the intestinal mucosa through M cells in Peyer’s patches, facilitating uptake by underlying macrophages, though it can also enter through enterocytes via a less efficient pathway [52, 53]. Once inside, MAP survives intracellularly and modulates the host immune response, allowing it to gradually spread to neighboring intestinal tissues and mesenteric lymph nodes, contributing to disease progression. In addition, MAP infection persistence for several years during the subclinical stage causing lesions mainly in intestinal tracts and related lymphoid tissues, progressively results in nutritional malabsorption, muscle wasting and lower productivity [5, 71, 72]. Understanding the molecular mechanisms of the host response to MAP infection at the primary sites of infection such as the JE, JELN and CPP will shed more light on the roles of biological molecules (such as miRNA) and the identification of biomarkers that may contribute to strategies to control the infection during the early stages. 

In this study the most miRNAs were expressed in the JE (448) followed by the JELN (410) and CPP (311) (Table S3). Some of the miRNAs were highly expressed in the JE (*n* = 28), JELN (*n* = 46) and CPP (*n* = 24) and functional enrichment of their target genes showed shared involvement in common immune processes such as immune response regulation, cell differentiation, and proliferation, with a particular focus on myeloid and lymphoid cell development, immune activation, and cytokine production (Table S4A-C). These processes are essential for maintaining immune homeostasis and responding to external stimuli. While JE emphasizes immune regulation pathways such as NF-kappaB signaling and phosphatidylinositol signaling, JELN further extends the functional spectrum by highlighting metabolic processes, including fatty acid and amino acid catabolism, along with DNA binding activities and stress responses. In contrast, CPP stands out with a strong emphasis on the regulation of the cell cycle, with terms related to the G2/M transition, sister chromatid segregation, and mitotic checkpoint regulation, underscoring its potential role in immune cell proliferation and differentiation. Additionally, JELN and CPP both highlight cellular responses to oxidative stress and mechanical stimuli, further differentiating them from JE. Together, these findings demonstrate the complex interplay between immune regulation, cell differentiation, metabolism, and stress responses across different biological contexts, providing valuable insights into the broader functional roles of miRNAs in these tissues.

Out of the observed highly expressed miRNAs, 23 demonstrated elevated expression in all tissues (Fig. 1), with bta-miRNA-143 standing out due to its exceptionally high levels. This miRNA has been extensively documented in various bovine tissues, including the intestinal tract, rumen, and skeletal muscle, thereby underscoring its vital regulatory functions in tissue development through its involvement in diverse cellular processes [73–76]. The abundant expression of bta-miR-143 has been previously associated with MAP infection [55, 77] and other bovine bacterial infections, such as mastitis [78, 79], highlighting its potential role in the host response to MAP infection. Additionally, bta-miR-145 was another miRNA with high expression across all three tissues. The synergistic effect of miR-145 and miR-143 has been implicated in inflammation and proto-oncogenes during gastrointestinal diseases, such as ulcerative colitis [80], colorectal [81] and gastric [82] cancers, suggesting that the interaction between bta-miR-143 and bta-miR-145 may play a regulatory role in lesion development in the gastrointestinal tract during MAP infection.

Different numbers of miRNAs were identified as DE in JE (*n* = 24), JELN (*n* = 35) and CPP (*n* = 49) across comparisons (Table S3F) and a similar trend was noted for enriched functional terms with more terms recorded for the CPP (1284 terms) followed by JELN (377 terms) and JE (4 terms). These differences may reflect the distinct roles of these tissues in the immune response and the varying degrees of mycobacterial uptake and persistence. The JE, being the first point of contact for pathogens, may have a more immediate, transient response to MAP infection, whereas JELN and CPP are more involved in sustained immune surveillance, which could explain the higher number of DE miRNAs and functional terms observed in these tissues. The increased number of DE miRNAs and functional terms in the JELN and CPP suggests that these tissues, which serve as key sites for immune activation and pathogen persistence, may undergo more complex regulatory changes in response to MAP infection. In contrast, the JE's role as a barrier may lead to a more limited miRNA response, focusing on initial defense mechanisms. This observation is supported by mRNA data which showed regionally distinct transcriptional responses in different gastrointestinal sites with more DE mRNAs reported in the ileum followed by JE, JELN and ileal lymph node as well as differences in the enriched immune and disease related pathways at these sites [34].

MiRNAs from several miRNA families were differentially expressed across tissues. The miR-2285 family, particularly bta-miR-2285f and bta-miR-2285i, were found to be highly expressed in all three tissues, including bta-miR-2285i being DE in MAPI vs HC comparison in JELN. The miR-2285 family has been implicated in immune responses and tissue-specific regulation during bacterial infections, highlighting its potential role in the pathogenesis of JD. For instance, the expression of miR-2285 family members were also identified in immune related tissues during bacterial infections, such as mammary epithelial cells [83], alveolar macrophages [84] and CD14 + monocytes [85]. The expression patterns of these miRNAs in the different tissues suggest that they may have tissue-specific regulatory functions, potentially modulating immune responses differently in the JE, JELN, and CPP. In the JELN, other DE miRNAs from the miR-2285 family, such as bta-miR-2285e and bta-miR-2285 m, were also identified, suggesting a broader involvement of this family in the immune response to MAP infection. In the JE, miRNAs such as bta-miR-2284 h-5p and bta-miR-2285e were DE, indicating their potential role in early immune responses. Meanwhile, in the CPP, miRNAs such as bta-miR-2285j and bta-miR-2285b were DE, further emphasizing the diverse roles of this miRNA family across tissues. Nevertheless, the exact regulatory mechanisms of the miR-2285 family in bovine diseases, especially JD, remain unclear and warrant further functional investigation.

Interestingly, six miRNAs (bta-miR-125a, bta-miR-146a, bta-miR-146b, bta-miR-21-5p, bta-miR-320a, bta-miR-370) showed dysregulated expressions (FDR < 0.1) in the JELN and CPP (Table 1) while four (bta-miR-146a, bta-miR-146b, bta-miR-2285e and bta-miR-122) showed dysregulated expression levels (*P* < 0.5 or FDR < 0.1) in all three tissues, suggesting roles in the host response to MAP infection. Out of these, bta-miR-146a, bta-miR-146b, bta-miR-21-5p, bta-miR-125a and bta-miR-320 were also reported as dysregulated in ileum and ileum lymph node tissues and bta-miR-370 in ileum tissue of the same cows in response to MAP infection [86]. Bta-miR-146a is known for its role in regulating immune responses, particularly by modulating the expression of pro-inflammatory cytokines such as TNF-α [87]. Its upregulation in all tissues in response to MAP infection supports its role in immune regulation. Meanwhile, the regulatory roles of bta-miR-146b in the immune system is often by interaction with bta-miR-146a [88, 89]. Consistently, upregulated expression of bta-miR-146a/b has been associated with other bacterial infection processes in dairy cows, such as *Mycobacterium bovis* infection and mastitis [90, 91], suggesting their important roles in the host immune response to MAP infection. Besides, bta-miR-146a was involved in the regulation of pathways related to specific immunity, such as the germinal center B-cell response and Th17 cell differentiation [92, 93]. The association of bta-miR-146a/b with immunity and bovine response to mycobacterial infection as well as their abnormal expression indicate their potential to be biomarkers for the management of MAP infection. Dysregulated expressions of bta-miR-122, bta-miR-125a and bta-miR-230a have been associated with viral and bacterial infections in cattle, chicken, pig, sheep and goat [94–97] while the miR-2285 family has been implicated in immune responses to livestock diseases. These observations support major roles for bta-miR-125a, bta-miR-146a, bta-miR-146b, bta-miR-21-5p, bta-miR-320a, bta-miR-370 in the immune response to MAP presence in the three gastrointestinal sites and their potential as biomarkers of MAP infection. In support of our findings, several studies have demonstrated the potential of miRNAs as biomarkers for MAP infection. For example, Shaughnessy and colleagues [51] identified three miRNAs from bovine feces capable of differentiating healthy cows from those with late-stage JD**.** Similarly, circulating miRNAs in bovine serum have been reported as important in the immune response to MAP or as biomarkers for early MAP infection [45, 46].

To better understand the biological significance of DE miRNAs, functional enrichment analysis was conducted for each tissue. The DE miRNAs identified in JE revealed enrichment in one immune and two metabolic pathways for the MAPI vs MAPT comparison only, hinting at a subtle or less involvement in large-scale functional alterations at the pathway level in JE during MAP infection.

DE miRNAs identified in JELN and CPP were enriched in many GO terms and KEGG pathways, indicative of possible regulatory roles during JD. First of all, the target genes of DE miRNAs in JELN and CPP were significantly enriched in many GO terms related to various metabolic and biosynthetic processes and the associated regulation, revealing that the abnormal expression of miRNAs in JELN and CPP may be a potential regulatory mechanism for the absorption and metabolic disorders causing malnutrition, emaciation and weight loss during JD. This observation is supported by observations with mRNA data and a metabolic reprogramming phenotype associated with JD [23, 34, 98]. Next, mickle cellular processes and development related GO terms were significantly enriched. For example, some enriched GO terms are important for fat metabolism (e.g. lipid, membrane lipid biosynthetic process, regulation of fatty acid biosynthetic process, fat cell differentiation) and organ development (limb, epithelium, muscle, vasculature, and heart). These enriched GO terms indicated the roles of DE miRNAs in JELN and CPP in some important developmental processes, which may be a possible explanation for the poor growth performance of MAP infected animals.

Meanwhile, the target genes of DE miRNAs in JELN and CPP were significantly enriched in more GO terms (e.g. lymphocyte activation, lymphocyte homeostasis, leukocyte differentiation, regulation of cell differentiation, T cell receptor complex, T cell selection, alpha–beta T cell activation, etc.) and KEGG pathways (e.g. T/B cell receptor signaling pathways, C-type lectin receptor signaling pathway and chemokine signaling pathway, etc.) involved in the innate immune and inflammatory responses, and disease processes [99, 100]. After MAP bacteria is phagocytosed by macrophages, it migrates to lymph nodes where it release antigens which stimulate T cells and B cells to start the host immune defense to MAP presence [53]. These observations suggest that DE miRNAs in JELN and CPP participate in the regulatory mechanisms of the host immune response to MAP infection. Furthermore, significantly enriched KEGG pathways related to virus infection, such as human cytomegalovirus infection and viral protein interaction with cytokine and cytokine receptor, as well as many KEGG pathways related to cancers and other diseases support important regulatory roles of DE miRNAs in JELN and CPP in the host immune response to MAP infection. Although JELN and CPP shared similarities in their functional enrichment, particularly in immune response pathways, JELN exhibited enrichments in pathways related to T cell activation and immune receptor signaling, while CPP showed a more diverse set of pathways, including those involved in Th17 cell differentiation and B cell activation. This suggests that JELN is critical for immune activation and regulation, whereas CPP may contribute more to adaptive immunity, particularly in the regulation of mucosal immune responses and the differentiation of immune cell subsets.

The comparison of MAPI to HC revealed a more pronounced immune response in JELN and CPP. This suggests that MAPI cows have a heightened immune response to MAP infection, likely due to the active presence of the pathogen (confirmed by immunohistochemistry and qPCR data [34]. In contrast, the comparison between MAPT and HC revealed fewer DE miRNAs and less pronounced immune activation. Moreover, the comparison between MAPI and MAPT revealed distinct differences in immune response activation. For instance, DE miRNA identified in CPP by MAPI vs MAPT showed enrichment for pathways related to Th17 cell differentiation, cytokine receptor activity, and JAK-STAT signaling. This suggests that MAPI cows experienced a more aggressive immune response compared to MAPT cows which might have developed strategies to contain the infection. Furthermore, out of 13 DE miRNAs common to MAPI vs HC and MAPT vs HC comparisons in CPP, four (bta-miR-484, bta-miR-21-5p, bta-miR-2285b and bta-miR-148a) had up-regulated expression in MAPI vs HC while five (bta-miR-125a, bta-miR-193a-5p, bta-miR-193b, bta-miR-382 and bta-miR-125b) had down-regulated expression in MAPT vs HC, implying that even the few DE miRNAs common to these phenotypes are expressed differently and so too will impact biological processes differently., supporting a role of miRNA in the ability of the host to tolerate the presence of MAP.

Recently, transcriptome profiling of MAP infected bovine macrophages was found to depict innate immune tolerance phenotypes [23]. Overall, tissue specificity was clearly demonstrated in the host response to MAP presence (summarized in Fig. 6). While majority of DE genes and functional terms were different for the MAPI and MAPT phenotypes, the few DE genes, enriched pathways and biological processes terms common to them were to different degrees. This demonstrates differences in the biological processes underlying these phenotypes and a role for miRNAs in the regulation of the transition in the host response from the presence of MAP bacterial (MAPI) to the development of tolerance (MAPT).

While this study provides valuable insights into the possible contribution of miRNAs to the regulation of MAP infection, it is important to acknowledge its limitations. Firstly, the small sample size and the use of DE miRNAs with corrected or uncorrected *p* values for functional analysis might have affected the reliability of the results generated. Therefore, further investigations with larger sample sizes are necessary to validate the DE miRNAs as well as the use of more advanced technologies to validate the functions of the DE miRNAs.

## Conclusion

More DE miRNAs were found in CPP (49) compared to JELN (35) and JE (24). Similarly, more enriched GO terms and KEGG pathways were found in the JELN and CPP compared to JE, showing tissue specificity in the miRNA expression and roles during MAP infection. Ten of the DE miRNAs were novel miRNAs identified in this study. Majority of enriched GO terms and pathways have immune and disease related functions suggesting participation in the regulatory mechanisms of the host immune response to MAP infection. The functional enrichments suggest a heightened immune response to MAP infection by MAPI group while the immune response by MAPT group was less pronounced suggesting that the MAPT cows have acquired tolerance to MAP infection. The JELN and CPP DE miRNAs were also significantly enriched in many GO terms and KEGG pathways related to various metabolic and biosynthetic processes suggesting metabolic reprogramming during JD. The biological processes underlying the MAPI and MAPT phenotypes were different or to different degrees. The miRNAs identified in this study (known and novel) will enrich the bovine miRNome and enhance knowledge about the potential of miRNAs in the host response to MAP infection. This study has provided further insights on the potential regulatory roles of DE miRNAs in the host response to MAP infection. The miRNAs commonly DE in JELN and CPP (bta-miR-125a, bta-miR-146a, bta-miR-146b, bta-miR-21-5p, bta-miR-320a, bta-miR-370) and those DE across all three tissues (bta-miR-146a, bta-miR-146b, bta-miR-2285e and bta-miR-122) have important regulatory roles during JD and could be potential biomarkers for the development of management solutions to control JD.

## Supplementary Information

Supplementary Material 1.

Supplementary Material 2.

Supplementary Material 3.

Supplementary Material 4.

Supplementary Material 5.

## Data Availability

The small RNA sequencing data generated during the current study are available in the National Center for Biotechnology and Information (NCBI) Sequence Read Archive (SRA) under accession number PRJNA1250568.
